# Technology-enhanced training in basic robotic surgical skills: a systematic review

**DOI:** 10.1007/s11701-026-03633-w

**Published:** 2026-07-20

**Authors:** Taner Shakir, Gita Lingam, Matthew Boal, Nader Francis, Manish Chand

**Affiliations:** 1 University College London, London, UK; 2 St Marks Academic Institute, Harrow, London, UK; 3 The Griffin Institute, Harrow, London, UK

**Keywords:** Robotic surgery, Simulation, Virtual reality, Augmented reality, Surgical education, Technology-enhanced learning

## Abstract

Robotic surgery has seen substantial growth, however training access remains limited by resources and time-intensive credentialling pathways. Technology enhanced learning may address these barriers whilst improving training efficiency and assessment objectivity. We aimed to systematically evaluate technology-enhanced approaches to basic robotic surgical skills training. This systematic review followed PRISMA guidelines with prospective registration (OSF: V93MZ). MEDLINE (via OVID), Embase, and the Cochrane Library (CENTRAL and CDSR) were searched on 23 May 2025 without date restrictions. Technologies included virtual reality, augmented reality, sensor-based systems, video review, and performance feedback. Risk of bias was assessed using the RoB-2 and ROBINS-I tools, and certainty of evidence using GRADE. Narrative synthesis was utilised given methodological heterogeneity, with reported outcomes mapped to the Kirkpatrick model of training evaluation. Seventeen studies involving 484 participants were included (10 randomised trials, 7 prospective cohort studies; 16 single-centre). Technologies comprised virtual reality (7), sensor-based systems (5), performance feedback (3), video review (1), and augmented reality (1); no eligible artificial intelligence studies were identified. Virtual reality, utilised within bespoke robotic simulators, demonstrated accelerated learning curves, although some systems required more repetitions than traditional robotic simulator-based training. The single augmented reality study improved instrument velocity. Sensor-based force measurement devices improved force application and ergonomics. Video review improved accuracy but temporarily increased completion time. Multimodal feedback systems outperformed conventional feedback. Evidence was concentrated at Kirkpatrick level 2 (learning); one study reported only level 1 (reaction) data, with no studies reporting level 3 or 4 outcomes. Risk of bias was low in randomised studies and low-moderate in non-randomised studies. Technology-enhanced basic robotic skills training has evidence supporting improved performance outcomes, accelerated learning curves, and enhanced objective assessment capabilities. Gaps persist for basic skills training in augmented reality, artificial intelligence, and docking and setup. Standardised competency assessment frameworks are required to evaluate these rapidly progressing tools.

**Supplementary Information:**

The online version contains supplementary material available at 10.1007/s11701-026-03633-w.

## Introduction

Robotic surgical procedures performed in 2024 were at least 2.7 million, with continued growth year on year [1]. Despite demonstrated benefits in patient outcomes and surgical ergonomics [2–4], global adoption remains limited by significant training barriers. Recent data has identified the negative impact of inequitable access and consultant learning curves leaving those training in robotic surgery disheartened [5, 6].

Current credentialling pathways are industry-driven and resource-intensive [7]. Training typically encompasses four phases: introductory online modules and assessments, pre-clinical simulation with time and score-based competency requirements, hands-on experience at centralised facilities, and proctored clinical cases [7–10]. This structure creates multiple bottlenecks, in part reflecting limited access to robots and simulators, as clinical service delivery demands frequently constrain the availability of systems for training [11–14]. Hands-on training and proctorship may further necessitate travel to centralised or, in some settings, international facilities, with resultant logistical, financial and environmental costs [15–18]. Progressing through not only basic skills and procedural training, but also progression from proctored to independent practice relies on subjective assessments rather than standardised metrics [19, 20]. The combination of these factors creates an inefficient, inequitable system that impedes robotics training.

The Royal College of Surgeons of England recently advocated for technology-enhanced training incorporating augmented reality (AR), virtual reality (VR), and artificial intelligence (AI) [9]. Such technologies could address access barriers through portable systems, objectify assessment through automated metrics, and accelerate learning curves through optimised feedback. However, evidence synthesis of these approaches in basic robotic skills training remains limited. This systematic review evaluated technology-enhanced interventions for basic robotic skills acquisition, focusing on their potential to overcome current training issues, and mapping reported outcomes against the Kirkpatrick framework [21] for evaluating training.

## Methods

This review was conducted and reported in accordance with the PRISMA 2020 statement and the PRISMA-S extension for reporting search strategies [22]. The protocol was prospectively registered on the Open Science Framework (ID: V93MZ) on January 23 2025.

### Eligibility criteria

Studies were included based on the following PICO format: the population comprised novice robotic surgeons; the intervention involved technology-enhanced basic robotic skills training; the comparator group was conventional basic robotic skills training methods; and outcomes included simulator scores, validated robotic assessment methods, automated performance metrics, or learning curves.

Technology-enhanced interventions were defined as adjunctive technologies working in conjunction with, or directly substituting for, conventional robotic basic skills training. Comparators were standard robotics skills and simulator-based training rather than a technology-free control, reflecting current practice in robotic-skills education. Basic robotic skills encompassed pre-procedural generic skills as defined in established literature [23, 24] which is the generic, pre-procedural competencies that precede procedure-specific training. Consistent with the phased framework described for UK robotic training [23, 24], basic robotic skills pertain to device-related skills (system set-up and docking) and basic console skills (camera and instrument manipulation), which are intended to be standardised and transferable across platforms and remain distinct from procedure-specific training. Exclusion criteria comprised studies lacking outcome assessment or measurable endpoints; studies focusing on procedure-specific rather than generic skills training; non-English language publications; and studies with irretrievable full text despite author contact attempts.

### Information sources and search strategy

A comprehensive search strategy was developed in collaboration with a medical librarian. Medical Subject Heading (MeSH) terms were combined with free-text keywords incorporating synonyms related to: robotic surgery (robotic surgical procedures, robot-assisted surgery), technology-enhanced learning (computer-assisted instruction, educational technology), simulation, virtual reality, augmented reality, artificial intelligence (machine learning, deep learning, neural networks), and three-dimensional video systems (stereoscopic video, 3D visualisation).

Four electronic databases were systematically searched on 23 May 2025: MEDLINE via OVID (1946 onwards), Embase (1974 onwards), Cochrane Central Register of Controlled Trials (CENTRAL), and Cochrane Database of Systematic Reviews (CDSR). No date restrictions were applied to the search strategy, and the search was restricted to English-language publications. Reference lists of included studies and relevant review articles identified during screening were hand-searched to identify additional eligible studies not captured by the electronic search. The full search strategy is provided in Supplementary Appendix S1.

### Study selection

Two reviewers (TS, GL) independently screened all titles and abstracts identified by the search strategy using standardised screening forms. Screening was conducted manually; no automation tools were used at any stage of study selection. Both reviewers independently assessed full-text articles against the inclusion and exclusion criteria. Disagreements at any stage of the screening process were resolved through consensus discussion with supervising authors (NF, MC). Reasons for exclusion at the full-text review stage were documented.

### Data collection

A standardised data extraction form was developed and piloted on three included studies before full extraction commenced. One reviewer (TS) extracted all data with verification by a second reviewer (GL). Extraction was performed manually using this form, without automated extraction tools. Disagreements in data extraction were resolved through discussion and reference to the original articles.

Extracted study characteristics included: publication year, country of origin, funding source or declared conflicts of interest (e.g. industry led), study design (randomised controlled trial, prospective cohort, retrospective cohort), number of participating centres (single-centre versus multicentre), total sample size, and participant expertise level (medical students, residents, fellows, or consultants).

Technological aspects extracted included: type (augmented reality, virtual reality, sensor-based, video review, or performance feedback), specific platform or device name, description of the technology, cost when reported, and whether the technology was commercially available or custom-built for research purposes. Intervention details extracted comprised: training duration, total training time, frequency of training sessions, specific tasks or exercises performed, supervision arrangements, and characteristics of the comparison group where applicable.

The primary outcome was performance on a validated composite assessment of robotic technical skill. This included objective, automated performance metrics and validated manual rating tools which quantify technical proficiency. Objective performance metrics encompassed kinematic data (e.g. instrument velocity, path length), force measurement, and other automated parameters (e.g. time-to-completion [TTC]). Validated manual rating tools included the Global Evaluative Assessment of Robotic Skills (GEARS) score, the Objective Structured Assessment of Technical Skills (OSATS), the Robotic Objective Structured Assessment of Technical Skills (R-OSATS), and other validated scoring systems. Secondary outcomes were also quantitative and comprised: simulator-derived scores (overall score, TTC, efficiency score, path length, penalty scores); learning-curve measures (repetitions to proficiency, CUSUM-derived plateau); perceived workload, assessed using the NASA Task Load Index (NASA-TLX); and reported cost. Remaining measures were qualitative or semi-quantitative and comprised Likert-based ratings of technology (e.g. depth perception, realism etc.), usability and acceptability ratings, semi-structured interview data, and open-ended feedback.

### Data synthesis

Substantial methodological heterogeneity was anticipated across included studies, encompassing differences in trainee populations, simulator platforms, intervention duration, comparator groups, outcome definitions, and study design. Furthermore, many studies utilised non-validated or technology-specific outcome measures, limiting meaningful statistical pooling. Thus meta-analysis was deemed inappropriate and narrative synthesis methods were employed. A systematic review was nonetheless considered the appropriate design, rather than a scoping review, because the review addressed a focused, pre-specified PICO question concerning intervention effectiveness and incorporated formal critical appraisal of risk of bias and of the certainty of evidence, rather than primarily mapping the breadth of the literature.

Results are presented as structured descriptive summaries organised by technology type. For each study, findings reported included group sample sizes, specific outcomes measured, summary statistics, and study conclusions. Data are presented in narrative form with supporting tables organised by technology and outcome to facilitate comparison across studies within technology categories. To characterise the level of educational impact addressed by each technology, reported outcomes were additionally mapped to the Kirkpatrick model of training evaluation [21].

### Risk of bias and certainty of evidence assessment

Risk of bias in randomised controlled trials was assessed using the Cochrane Risk of Bias tool version 2 (RoB 2), and non-randomised studies were assessed using the Risk Of Bias In Non-randomised Studies of Interventions (ROBINS-I) tool. Two reviewers (TS, GL) independently conducted all risk of bias assessments. Disagreements were resolved through consensus discussion. When consensus could not be reached, a third reviewer (MB, NF or MC) adjudicated. The robvis visualisation tool [25] was used to generate summary plots and traffic light plots displaying risk of bias assessments across studies. Certainty of evidence for each outcome domain was appraised using the Grading of Recommendations, Assessment, Development and Evaluations (GRADE) framework, with judgements presented in Supplementary Appendix S2.

## Results

**Fig. 1:**
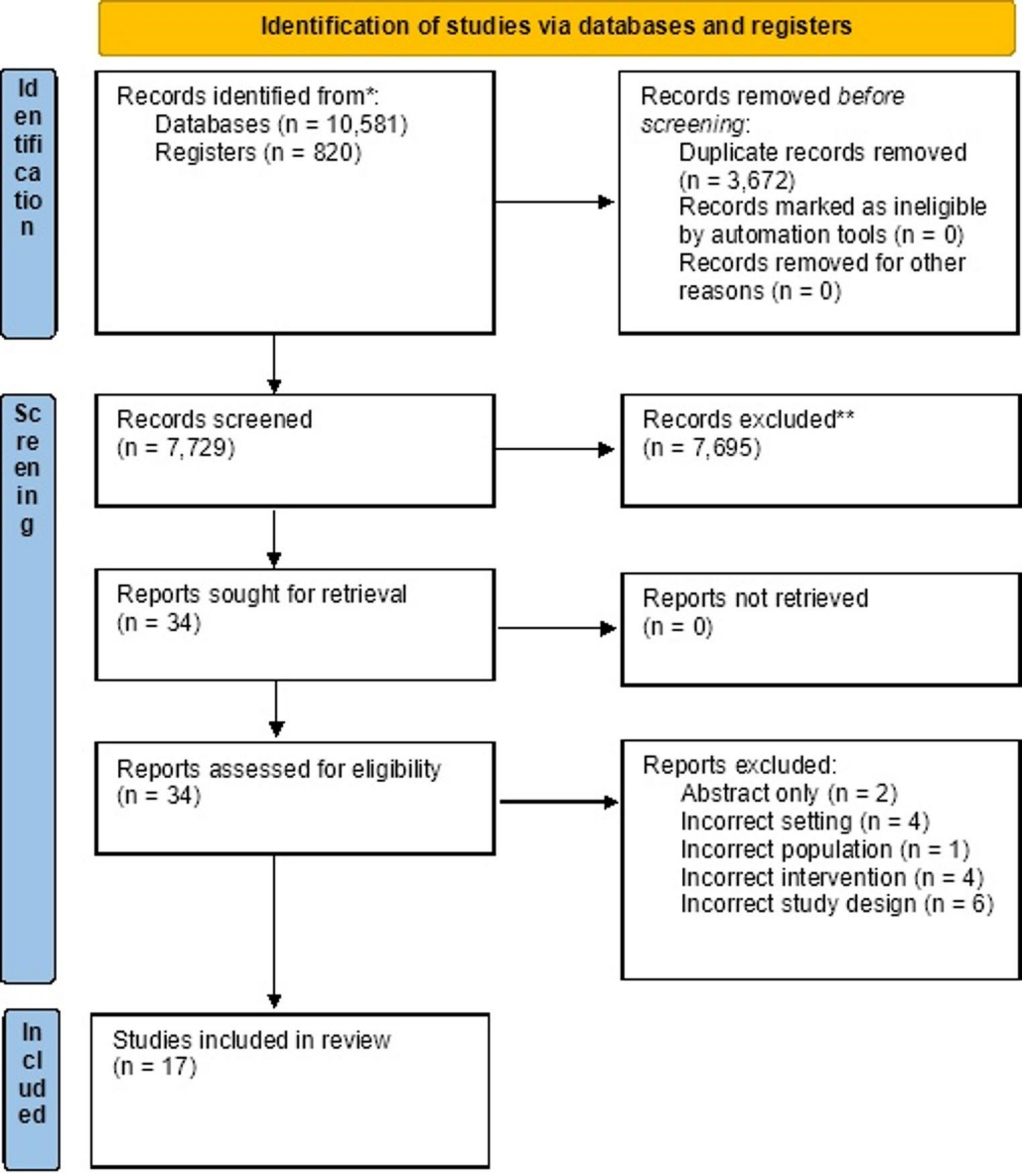
PRISMA 2020 Flow Diagram

The search identified 11,401 records, of which 3,672 duplicates were removed; 7,729 records were screened at title and abstract level (Fig. 1). Seventeen studies met the inclusion criteria (Table 1). No additional studies were identified through reference screening.

There were 484 total participants over the 17 studies, with technologies including VR (*n* = 7), AR (*n* = 1), sensor-based (*n* = 5), video review (*n* = 1), and performance feedback (*n* = 3). There were 10 randomised trials (including one randomised crossover) and 7 prospective cohort studies; sixteen studies were single-centre, with one multicentre. No studies evaluating artificial intelligence, machine learning, or deep learning met the inclusion criteria. Mapped against the Kirkpatrick framework for evaluating training, sixteen studies reported level 2 (learning) outcomes, comprising simulator-derived scores, kinematic or force-based metrics, or validated rating tools. One single-arm study reported level 1 (reaction) data only, in the form of Likert-based usability ratings. No study reported level 3 (transfer to operative behaviour) or level 4 (patient-level) outcomes. Study-level assignments are presented in Table 1.

**Table 1:** Characteristics and outcomes of included studies (*n* = 17)

Author, Year	Country	Design	*n*	Population	Technology Category	Intervention	Comparator	Tasks / Setting	Outcome Measures	Findings	COI	KP level
**Virtual reality (n = 7)**												
Cai, 2023	China	Prospective cohort	25	10 experts; 15 novices	Virtual reality	Virtual Reality Digital Twin (VRDT): bespoke haptics-enabled robotic console with VR simulation	Expert vs. novice (construct validity)	Peg transfer; soft tissue cutting	Total time; movement length; peg drops; cutting frequency	Experts outperformed novices on peg transfer for time-to-completion (TTC) and right/left instrument movement (*p* = 0.00002, *p* = 0.03, *p* = 0.04); differences more pronounced on soft tissue cutting (*p* < 0.01 across all domains)	Nil	2
Feifer, 2011	USA	RCT	20	Medical students	Virtual reality	Hybrid VR simulator with motion capture (LapSimVR + ProMIS)	Hybrid training; conventional training; combined; no training	FLS-adapted basic skills tasks (peg transfer, cutting, intracorporeal knot, cannulation) on da Vinci Skills Simulator (dVSS)	Validated normalised composite score (4 domains)	Hybrid group: 27.9% improvement in normalised score (*p* = 0.03); no significant change in conventional-only group	Nil	2
Chien, 2012	USA	RCT	14	Medical students	Virtual reality	Portable VR simulator with custom training environment and portable hand controllers (40 min) vs. 3D tennis game (40 min)	3D video game	Bimanual carry; peg transfer on dVSS	Robotic kinematic data — economy of movement; total distance (mm); TTC	VR group: 15.0% improvement in economy of movement vs. 0.63% increase in distance in 3D-game arm (*p* < 0.001); VR significantly faster TTC (*p* = 0.002)	Nil	2
Hardon, 2022	Netherlands	RCT	38	Medical students	Virtual reality	Portable Laparoscopic Robotic Surgery (PoLaRS) simulator + ForceSense (7 degrees of freedom; force sensors)	dVSS only	PoLaRS exercises (marble sorting, pick and place, pass through); dVSS instructional content	TTC; path length; force application	Both groups improved significantly during training; no between-group differences at baseline or post-training for TTC, path length, or force application	Nil	2
Casas-Yrurzum, 2023	Spain	Prospective cohort	15	Robotic surgery masters students	Virtual reality	Web-based stereoscopic 3D viewer with virtual annotation/telestration capability	None (single-arm usability evaluation)	1-hour teacher-led review of robotic mediastinal cyst resection	Bespoke 15-item Likert questionnaire (depth perception, annotation, telestration, segmentation, usefulness)	Median scores 6/7 or 7/7 across all domains; all domains showed significant improvement vs. neutral (*p* < 0.001)	Nil	1
Nathan, 2023	UK	Randomised crossover	11	Novice surgical trainees	Virtual reality	BARCO WeConnect virtual classroom — interactive VR-based technical and non-technical skills tutorials (1 week per arm)	Self-directed Fundamentals of Robotic Surgery (FRS) learning	FRS simulator tasks	Robotic-Objective Structured Assessment of Technical Skills (R-OSATS)	VR-classroom arm: significantly higher R-OSATS (44.80 vs. 35.33 points, *p* = 0.006)	Nil	2
Eley, 2024	UK	RCT	23	Medical students	Virtual reality	Bespoke VR e-learning modules for operating room/console set-up (*n* = 11)	Conventional e-learning (*n* = 12)	Live setup task assessed by two blinded raters across 6 modules (moving/preparing surgeon console, instrument/visualisation bedside connection, draping, instrument identification/attachment, port training, surgical mode entry)	5-point Likert intervention scale (1 = independent → 5 = physical assistance); time taken	VR arm required less rater intervention (53.5 vs. 84.5, *p* < 0.001); no time difference (22 vs. 22.5 min, *p* = 0.880)	Industry led	2
**Augmented reality (n = 1)**												
Judkins, 2008	USA	Prospective cohort	30	Medical students	Augmented reality	Real-time AR visual overlays within console (speed, grip force, or relative inter-instrument movement)	Three sub-groups receiving different AR overlays	Bimanual carrying; needle passing; suture tying on dVSS	TTC; instrument velocity; distance; curvature; grip force; relative phase — measured immediately post-training and at 2-week retention	AR-speed sub-group: mean instrument velocity nearly doubled in bimanual carrying (21 → 38 mm/s, *p* < 0.05); gains retained at 2 weeks	Nil	2
**Video review (n = 1)**												
Takagi, 2023	Japan	RCT	20	Surgical trainees	Video review	3-hour ‘tips and tricks’ educational videos covering 9 dVSS tasks (*n* = 10)	No video, no instruction (*n* = 10)	9 dVSS tasks × 10 cycles (sea spikes 1 & 2, camera targeting 1, suture sponge 1, thread the ring, energy switching 1, ring and rail 1, needle targeting, 30° scope swap)	Overall score; efficiency score; penalty score; CUSUM analysis of TTC learning curve	Mean overall score 90.8 vs. 72.4 (*p* < 0.001); video group plateaued at 2 cycles vs. 4 cycles in control	Nil	2
**Sensor-based (n = 5)**												
Yang, 2017	France	RCT	20	Medical students	Sensor-based	Pressure-surveillance armrest with audible alarm when forearms left armrest contact	No alarm (conventional armrest)	Peg board 2; match board 2; thread the ring 1 — 15 attempts each on dVSS	dVSS simulator scores; bespoke armrest load score	Alarm group: higher simulator scores (*p* < 0.05); better armrest load score (*p* < 0.001)	Nil	2
Takayasu, 2018	Japan	Prospective cohort	20	10 experts (> 100 cases); 10 novices (medical students)	Sensor-based	OptiTrack Flex 3 optical motion tracking of upper-body posture	Expert vs. novice (construct validity)	Suture sponge 1; tubes	Joint angles, elbow/wrist position relative to armrest; dVSS automated metrics	Novices: greater elbow/wrist extension (> 50°), higher right-elbow position, lower right-wrist position relative to armrest (*p* < 0.001); decreased simulator scores in 8/9 metrics	Nil	2
Wu, 2021	USA	Prospective cohort	7	Novice urology trainees	Sensor-based	Portable EEG and Tobii Pro Glasses 2.0 eye-tracking; engagement index, gaze entropy, pupil diameter	None (longitudinal observational)	12 dVSS exercises across 26 sessions over 3 months (camera targeting, peg board, ring and rail, sponge suturing, dots and needles, tubes)	Engagement index; pupil diameter; gaze entropy; NASA-TLX; simulator performance scores	Increasing simulator scores correlated with decreased engagement index and gaze entropy (*p* < 0.001); 72.5% prediction accuracy for performance	Nil	2
Rahimi, 2023	Netherlands	Prospective cohort	60	20 novices; 20 intermediates; 20 experts	Sensor-based	ForceSense pressure sensor capturing applied force during suturing	Cross-skill comparison (construct validity)	Robotic suturing — 6 trials per participant (360 trials)	TTC; mean non-zero force; maximum impulse; force volume	Experts faster (22 s vs. 41 s, *p* = 0.003) and applied lower force (19 N vs. 29 N, *p* = 0.032) than novices; tool discriminated skill levels (*p* < 0.05)	Nil	2
von Bechtolsheim, 2024	Germany	RCT	87	Medical students; first-year residents	Sensor-based	ForceTrap^®^ three-dimensional force-measuring device combined with VR (*n* = 43) or live console FRS training (*n* = 44)	VR vs. live console (head-to-head)	FRS dry-lab tasks (flap, precise cut, dissection, suture); VR FRS proficiency tasks (Ring Tower Transfer, Knot Tying, Vessel Energy Dissection)	Mean non-zero force; peak force; TTC; repetitions to proficiency	Both arms reduced peak force; no significant final force difference (suturing peak force: console 4.1 N vs. VR 4.7 N, *p* = 0.086); console arm faster at midpoint (Flap 59.2 vs. 73.4 s, *p* = 0.007; Suture 183.8 vs. 227.2 s, *p* = 0.006); fewer repetitions to proficiency on Ring Tower (2.48 vs. 5.45, *p* < 0.001), Knot Tying (5.34 vs. 8.13, *p* = 0.006), Vessel Energy Dissection (2.0 vs. 2.38, *p* = 0.001)	Nil	2
**Performance feedback (n = 3)**												
Yang, 2017	France	RCT	60	50 surgeons; 10 nurses	Performance feedback	Controller of Events on Simulator and Robot (CESIR) — multimodal cameras + force/pressure sensors enabling retrospective visualisation of hand-foot kinematics and armrest contact	Conventional feedback	Match board 2; thread the rings 1 — 10 repetitions each	dVSS simulator scores	CESIR group improvement 64 → 91 vs. conventional 63 → 79 (*p* < 0.05)	Nil	2
Abdelaal, 2019	Canada	Prospective cohort	12	Not reported	Performance feedback	Additional intracorporeal camera providing dual viewpoint within console viewfinder	Single (conventional) view	Ring and rail dry-lab task — 10 repetitions	Completion time; error time; retention and transfer-task accuracy	Dual-view group: 57% greater accuracy on retention; 35% greater accuracy and 25% faster on transfer task vs. single-view	Nil	2
Postema, 2024	Netherlands	RCT	22	Residents	Performance feedback	Updated PoLaRS simulator with real-time haptic (controller vibration) and visual (red flash) collision feedback (*n* = 11)	No feedback (*n* = 11)	VR pick-and-place; 6 trials × multiple tasks (198 total trials)	TTC; tip-to-tip distance; path length; collision count	No overall between-group performance differences; feedback group: fewer dominant-hand collisions (8.73 vs. 14.27, *p* = 0.045)	Nil	2

COI: Conflict of Interest; AR, augmented reality; CESIR, Controller of Events on Simulator and Robot; CUSUM, cumulative sum; dVSS, da Vinci Skills Simulator; FLS, Fundamentals of Laparoscopic Surgery; FRS, Fundamentals of Robotic Surgery; KP, Kirkpatrick training-evaluation level; NASA-TLX, NASA Task Load Index; PoLaRS, Portable Laparoscopic Robotic Surgery; R-OSATS, Robotic-Objective Structured Assessment of Technical Skills; RCT, randomised controlled trial; RoB 2, Cochrane Risk of Bias tool 2; ROBINS-I, Risk of Bias in Non-randomised Studies of Interventions; TTC, time-to-completion; VR, virtual reality; VRDT, Virtual Reality Digital Twin

### Virtual reality

A custom built virtual reality simulator was developed using VR glasses and haptics enabled hand controllers [26]. The simulation aspect was a bespoke design, with two tasks modelled – peg transfer and soft tissue cutting. A separate recording device captured instrument motion and TTC. A group of 15 novices and 10 experts were divided, with novices receiving a training session prior to completion of the two tasks. Experts performed significantly better on the peg transfer task with respect to TTC, right and left instrument movements (*p* = 0.00002, *p* = 0.03, *p* = 0.04 respectively). This was more pronounced on the soft tissue cutting task (*p* < 0.01 for all domains).

Another VR based hybrid simulator was developed with additional instrument motion capture abilities [27]. This was evaluated as a means of subsequently improving robotic console performance. Twenty medical students were randomised to receiving hybrid training compared with conventional training, a combination of the two, or no training. There was a 27.9% (*p* = 0.03) improvement in a validated normalised score adapted from Fundamentals of Laparoscopic Surgery (FLS), when performing 4 basic skills tasks, within the hybrid group. There was no statistically significant difference in the conventional training group.

VR was utilised in the operating room setup of a robotic platform [28]. Custom built VR e-learning modules provide immersive simulation of setup in a virtual operating room. Twenty three medical students were randomised to either VR learning (*n* = 11), or conventional e-learning (*n* = 12). Subjects were assessed by blinded raters using a subjective rating tool. The VR group required less intervention (53.5 vs. 84.5, *p* < 0.001), whereby a 5 point Likert scale was used with 1 indicating independent performance and 5 was requiring physical assistance. There was no statistically significant difference in TTC with respect to the e-learning group (22 vs. 22.5 min respectively, *p* = 0.880).

A portable VR simulator was investigated compared with playing a 3D game prior to performing basic robotic skills tasks [29]. The simulator had portable hand controllers with a custom made VR training environment. 14 medical students were randomised to one of the technologies for 40 min before performing bimanual carry and peg transfer tasks. Assessment was through robotic kinematic data, with the VR group displaying a 15.0% improvement in economy of movement compared with a 0.63% increase in total distance travelled (mm) in the 3D game group (*p* < 0.001). There was also significant improvements in TTC in the VR group (*p* = 0.002).

An RCT was conducted with the aim to validate a different form of virtual reality simulator termed the Portable Laparoscopic Robotic Surgery (PoLaRS) simulator [30]. This device consists of a console and two handles, which mimic the seven degrees of freedom of the robotic systems. Force sensors were applied to the instruments to assess pressure. 38 medical students were randomised to training on only the da Vinci Skills Simulator (dVSS), or both the dVSS and PoLaRS. Whilst each parameter within both arms improved significantly throughout the study, there were no differences between the two groups at baseline or after training for TTC, path length, or force application.

A novel VR based tool with a stereoscopic viewer was developed [31]. This web-based application allows for observation in 3D, with the added ability of being able to annotate virtual information onto the video. This was tested in a teacher-student environment, with 15 robotic surgical masters students shown a robotic mediastinal cyst resection with one teacher guiding learning. After a one hour teaching session, users completed qualitative feedback of a bespoke 15 point questionnaire with Likert scales. Questions centred on depth perception, annotations and overall impression. Median scores were either 6 or 7 across all domains on a 7 point Likert scale, with all domains having a significant improvement from neutral (*p* < 0.001).

Adjunctive learning utilising a VR headset was used to assess Fundamentals of Robotic Surgery (FRS) simulator tasks [32]. A randomised crossover study included 11 novice surgical trainees, who were allocated to either self-directed learning, or adjunctive interactive VR classroom training. After receiving either learning intervention for one week, the groups crossed over for another week. Assessment of simulator task performance was using the validated Robotic-Objective Structured Assessment of Technical Skills (R-OSATS) at the end of each week. The VR group achieved significantly higher scores compared with self-directed FRS alone (44.80 vs. 35.33 points, *p* = 0.006).

### Augmented reality

One study utilised AR with augmented visual feedback [33]. This allowed real time overlays of performance including indicators of speed, grip force and relative movements of left and right instruments. This feedback was shown whilst performing three simulator tasks (bimanual carrying, needle passing, suture carrying). Thirty novice users were divided into groups where they receive one of the aforementioned indicators. Performance was assessed using TTC, and kinematic parameters immediately post-training, and at a two-week retention test. Greatest improvements were noted in the AR speed group, whereby mean instrument velocity almost doubled in a bimanual carrying task from 21 to 38 mm/sec (*p* < 0.05). These improvements were noted to remain at interval testing after 2 weeks.

### Video review

Educational videos of robotic skills were evaluated as a means of improving simulator scores [34]. This involved 3-hour tips and tricks videos of the nine simulator tasks which were about to be performed. 20 participants were randomised into a video (*n* = 10) and control (*n* = 10) group. Participants performed nine tasks in one cycle, and repeated this 10 times. Performance was evaluated using simulator metrics including overall score, efficiency score and penalty score. Mean overall score was 90.8 vs. 72.4 in the video and control groups respectively (*p* < 0.001). CUSUM analysis of the learning curve for TTC reported the video group plateaued within 2 cycles, with the control group plateauing at 4 cycles.

### Sensor based

An alarm based armrest was evaluated [35]. This encompassed a pressure surveillance system utilising force sensors. Alarms were triggered when the user’s forearms were not in contact with the armrest. Subjects were divided into two groups, with one group performing simulator tasks with the alarm, and the other group without. Both groups had their armrest load scores evaluated, and performed 3 simulator tasks (peg board 2, match board 2 and thread the ring 1). Twenty novices were randomised to 15 attempts at each task, with the alarm group having significantly higher simulator scores (*p* < 0.05). Furthermore, a bespoke armrest load score was calculated, indicating the alarm group had better robotic console ergonomics (*p* < 0.001).

Posture was assessed during simulator tasks by an optical motion tracking system [36]. An observational study of 10 experts and 10 novices evaluated upper body motion during 2 tasks (suture sponge 1, tubes). Novices had significantly more elbow and wrist extension (> 50°), higher elbow heights, and lower wrist positions relative to the armrest on their right hands (*p* < 0.001). This was in line with significantly decreased simulator scores across 8/9 automated simulator parameters.

A prospective study analysed 7 novice urology trainees with respect to simulator performance [37]. Portable EEG monitoring and eye tracking was used to measure cognitive workload. Participants then performed 12 robotic simulator exercises. An engagement index was calculated from a ratio of brainwave activity, with a higher score meaning more cognitive engagement. Alongside this, a gaze entropy score was calculated based on eye movements and pupil diameter, with increased pupil diameter and gaze movements indicative of increased cognitive workload. Correlation was noted between increasing simulator scores and decreased engagement index and gaze entropy (*p* < 0.001). The authors noted that these workload metrics correlated with training performance (reported predictive accuracy 72.5%), although this finding is exploratory and reflects cognitive workload rather than operative skill.

Analysis of force was utilised during suturing skills as a means of differentiating between varying skill levels [38]. ForceSense, a force measuring system, provides information on mean and maximum force applied, in addition to TTC. This was tested on a total of 60 participants (20 novices, 20 intermediates and 20 experts) performing 6 suturing trials. A total of 360 trials were performed, with significant differences observed between novices and experts. Experts completed tasks quicker (22s vs. 41s, *p* = 0.003), and used lower force (19 N vs. 29 N, *p* = 0.032), reflecting gentler tissue handling.

Similar technology was also utilised to assess whether learning on VR or on the robotic system itself made a difference to force applied [39]. A prospective RCT (*n* = 87) compared basic skills learning in novices (medical students and residents) randomised into two groups – one on the VR simulator (*n* = 43), and another on the system itself (*n* = 44) performing dry lab FRS tasks at two time points. Tissue handling was assessed using ForceTrap^®^ - a force-measuring device capturing force application in three-dimensions. Both groups achieved significant reductions in peak force application across all tasks. However, there were no significant differences in final force outcomes between groups (e.g. peak force in final suturing task: robotic console 4.1 N vs. VR 4.7 N, *p* = 0.086). Despite this, the robotic console group had faster TTC at midpoint testing (Flap: 59.2s vs. 73.4s, *p* = 0.007; Suture: 183.8s vs. 227.2s, *p* = 0.006). Furthermore, the robotic console group needed fewer training repetitions to achieve proficiency in three VR FRS tasks: Ring Tower Transfer (2.48 vs. 5.45, *p* < 0.001), Knot Tying (5.34 vs. 8.13, *p* = 0.006), and Vessel Energy Dissection (2.0 vs. 2.38, *p* = 0.001).

### Performance feedback

A multimodal method of providing individualised feedback using a combination of cameras and force sensors was termed Controller of Events on Simulator and Robot (CESIR) [40]. This allows the user to retrospectively visualize how their hand and feet movements correlate to simulator performance, with the additional visual information of when the users arms were off the armrest, via a pressure sensor. A randomized study of 60 robotic novices were divided into a group receiving CESIR feedback (*n* = 30), and a group with conventional feedback (*n* = 30), after performing 2 simulator tasks 10 times (match board 2, thread the rings 1). Simulator scores showed a greater improvement in the CESIR group from 64 to 91, whereas the conventional group improved from 63 to 79 (*p* < 0.05).

An additional intracorporeal camera was postulated to aid in improving training in a ring and rail dry lab skills task [41]. This camera, placed at a different angle within the training environment, allows an additional view to be displayed in the consoles viewfinder. Twelve subjects were randomly assigned to the dual or single (conventional) view. Basic skill task accuracy was improved by up to 57% in the dual view group, in addition to a 25% improvement in TTC for a transfer task.

A newer version of the aforementioned PoLaRS simulator was utilised with a different technological aspect – this time in the context of additional visual and haptic feedback [42]. Simulator tasks in VR were performed, with collisions resulting in vibrations from the hand controllers, in addition to a red colour being displayed on screen. Novices were randomised into two groups – one which received haptic and visual feedback (*n* = 11), with the other group receiving no feedback (*n* = 11). A total of 198 tasks were performed, with no overall improvements between the groups. The visual and haptic feedback group, however, exhibited fewer collisions with their dominant hands (8.73 vs. 14.27, *p* = 0.045).

### Risk of bias and certainty of evidence assessment

**Fig. 2:**
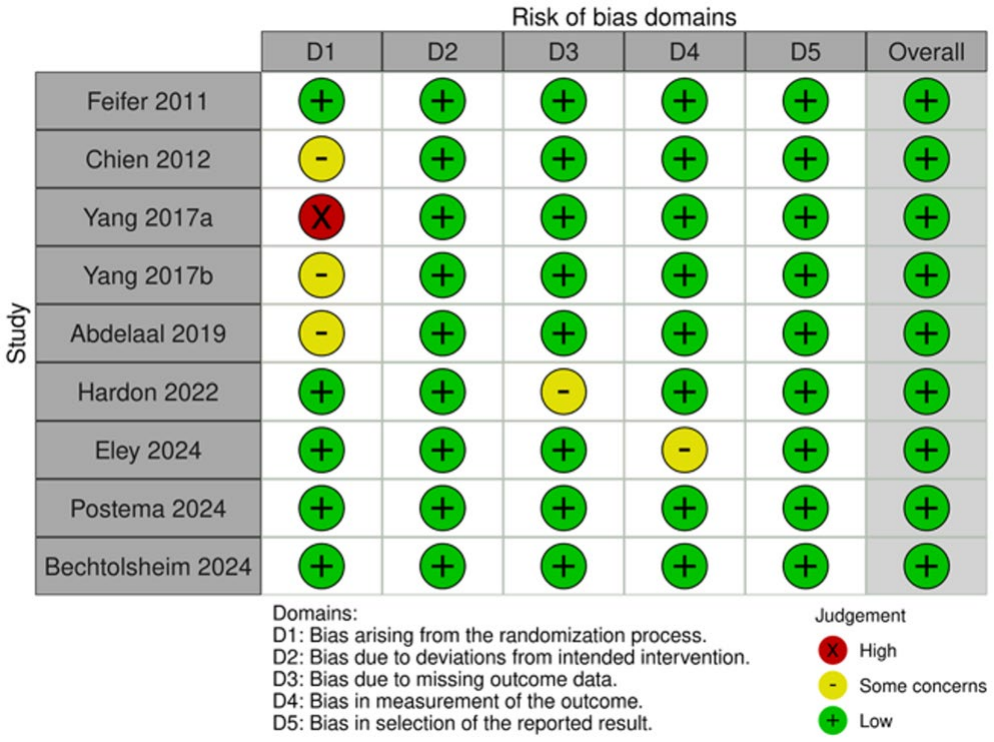
Traffic light plot for risk of bias of randomised studies

The majority of RCTs had low overall risk of bias (Fig. 2). For these studies, most concerns related to the randomisation process or outcome measurement. One study showed high risk regarding randomisation but maintained low overall risk.

**Fig. 3:**
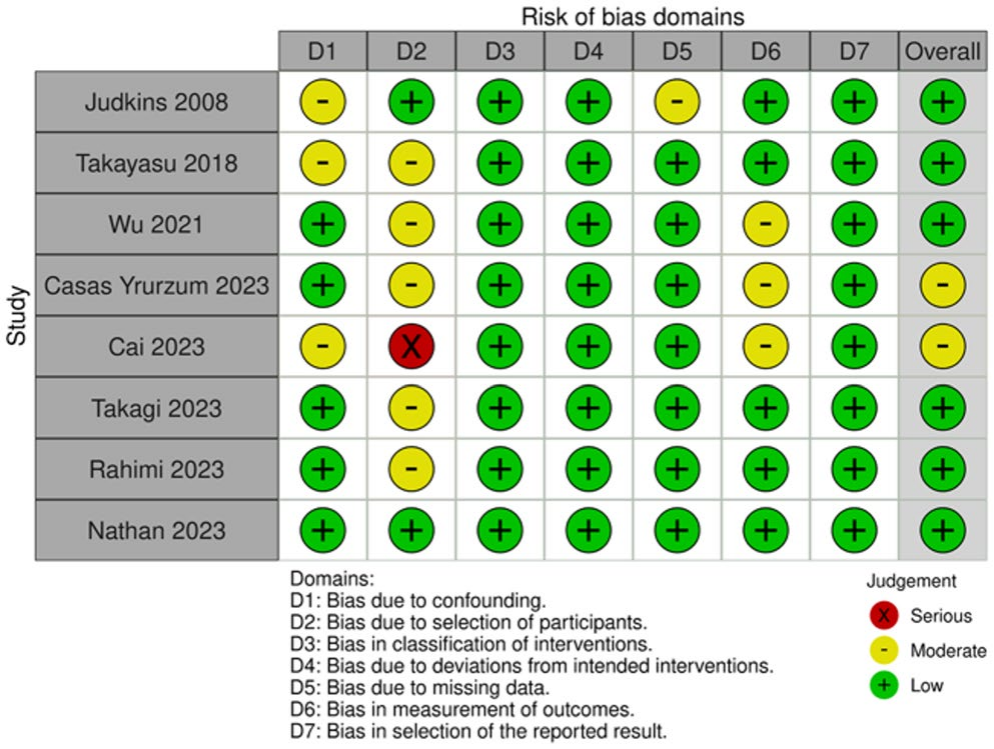
Traffic light plot for risk of bias of non-randomised studies

For non-randomised studies, bias risk for the majority was low-moderate, with common concerns relating to confounding and outcome measurement. One study was judged at serious risk regarding participant selection (Fig. 3).

Applying the GRADE framework (Supplementary Appendix S2), the certainty of evidence was low for composite simulator score, force application, and learning-curve outcomes, and very low for TTC, kinematic precision, validated rating-tool scores, skill retention, and user acceptability. No included study provided evidence on operative-skill transfer or patient-level outcomes.

## Discussion

This review evaluated technology-enhanced approaches to basic robotic skills training across 17 studies involving 484 participants. Overall findings were that various technological adjuncts can effectively enhance conventional training, while concomitantly revealing evidence gaps. This included VR systems achieving simulator score improvements in addition to halving the time to reach the learning curve plateau. AR trained individuals were reported to have instrument velocity improvements, and feedback systems demonstrated collision reduction. This was generally in studies with a low risk of bias, however, with a low- to very low certainty of evidence.

The ability of technology enhancement to provide, and improve objective metrics including position, speed, accuracy, force application, and error reduction, represents an opportunity to enhance training. Each technology type was evaluated against respective components identified during this review, and summarised in Table 2. Mapping these findings to the Kirkpatrick model [21], the available evidence is concentrated at level 2 (learning — simulator scores, kinematic metrics, R-OSATS), with one study level 1 (learner reaction), and with no included study reporting level 3 (transfer to operative behaviour) or level 4 (patient outcomes); this limits extrapolation of the findings to clinical performance.

**Table 2:** Technology Summary

Technology Type	Speed/Efficiency	Accuracy/Error Reduction	Learning Curve	Objective Metrics	Cost-Effectiveness	Accessibility	Evidence Base
**Virtual Reality**	✓	✓	✓✓	✓	?	✓✓	Moderate
**Augmented Reality**	✓	✓	✓	✓	?	✓	Low
**Sensor Based**	✓	✓✓	✓	✓✓	?	✗	Moderate
**Video Review**	?	✓	✓	?	✓	✓✓	Low
**Performance Feedback**	✓	✓	✓	✓	?	?	Low-Mod

**Key**: ✓ = Supported; ✓✓ = Strongly supported; ✗ = Not supported; ? = Insufficient data

Virtual reality was the most studied modality, likely reflecting its established role and use in simulation for robotic skill acquisition, and the relative ease of developing bespoke virtual-reality environments for investigation, with seven studies showing accelerated learning curves. A combination of custom built simulators or novel technology aimed at improving the learning within the basic skills sphere were employed with significant findings. However, in some instances VR enhanced training required more repetitions than conventional training to achieve equivalent proficiency, with some cases being more than double. It should also be mentioned that the majority of the virtual reality based technologies were custom built and therefore non-commercially available, this would limit the scalability and reproducibility. However, with refinements and commercialisation, it may be that VR could be employed as an adjunct to aid the initial learning curve, especially when there is resource and access limitations with respect to robotic consoles. Headsets are able to be used from the comfort of one’s own home, which could accelerate learning when able to access the system.

Sensor-based technologies demonstrated validity, with force measurement able to differentiate experts from novices. Promising technological advances included armrest alarms and ergonomic improvements. These utilised quantifiable objective metrics, which have the potential be able to determine competency thresholds and reduce subjectivity. Neurocognitive monitoring using EEG and eye-tracking correlated with training performance (an exploratory finding reflecting cognitive workload rather than operative skill), raising possibilities for systems that could regulate task difficulty based on real-time cognitive workload.

Video review was an interesting method utilised to aid with improving the initial simulation component. Findings from a small scale study showed improved technical accuracy but longer TTC, likely reflecting the transition from unconscious to conscious incompetence—a necessary developmental stage rather than training failure. Multimodal performance feedback systems achieved superior improvements compared with conventional feedback, suggesting feedback approaches addressing multiple domains are more effective than isolated metrics.

Only one study utilised augmented reality, possibly reflecting the more limited commercial availability of mature AR headsets at the time of the included studies. Nonetheless, results showed promise with improvements in kinematic parameters such as instrument velocity, which persisted after a time interval. Unlike the complete immersion experience in VR, AR overlays instructional information onto ones vision, potentially negating any side effects associated with headsets such as dizziness or nausea.

These findings broadly align with prior syntheses of robotic-surgery training [43–45], which identified VR simulation as the dominant modality and noted evidence gaps in standardised assessment, clinical correlation, and cost. The present review goes further by highlighting sensor-based and feedback-driven adjuncts. A further recent systematic review of robotic training modalities and assessment methods similarly reported a predominance of dry-laboratory and virtual-reality simulation and the common use of GEARS and OSATS, while noting that focused training in tissue handling and force application remains scarce despite the absence of haptic feedback [13]; this concurs with our sensor-based and force-measurement findings.

Portable VR systems can be transported relatively easily compared with current robotic simulators, potentially improving access on a global level. Current training pathways often require extensive travel to national or international facilities [15, 20]. The associated travel may carry a substantial carbon footprint [46], although the magnitude of this effect for surgical-training programmes has not been formally quantified. Portable simulation and video-based remote assessment could offer a means to reduce travel and time commitments. In addition, clinical systems are increasingly used after hours and on weekends [47]. Finding time when a simulator is available in one’s institution can be extremely challenging. Adjuncts to aid with skill acquisition should be explored.

### Limitations

Several limitations were present. Sixteen of seventeen studies were single-centred, and sample sizes were uniformly small (median 20, range 7–87); this combination limits statistical power, restricts external validity, and the strength of inferences that can be drawn. Certainty of evidence, with respect to outcomes, was low to very low. Studies mainly addressed console skills, with only one trial examining setup processes, which was limited by subjective ratings. A reliance on medical students was also observed, which may not reflect surgical resident, or consultant learning curves. As aforementioned with relation to the Kirkpatrick model, no study examined whether simulator improvements translate to superior operative performance or patient outcomes, leaving clinical relevance uncertain, though this likely reflects the exploratory nature of these initial trials. Furthermore, economic implications were not examined, with only minimal cost reporting. True benefits may be underestimated given potential savings through negating travel requirements.

Retention of skill was rarely measured (only one trial reported a 2-week retention test), limiting inferences regarding durability of training gains. Time-to-completion, the most frequently reported outcome, may favour a faster but less precise participant performance, and therefore may limit interpretation. The most utilised system was from the market leading supplier, whereby the findings may not be transferable to other robotic platforms. Finally, all included studies originated from high-income settings, which constrains the generalisability in the global health setting.

Limitations also apply to the review process itself: synthesis was narrative rather than meta-analytic, given the clinical and methodological heterogeneity across interventions and outcomes, which precluded quantitative pooling. The search was limited to MEDLINE, Embase and the Cochrane Library and to English-language reports identified on a single date (23 May 2025), and data extraction was undertaken by one reviewer with another reviewer independently verifying; the possibility of language and publication bias, and of relevant studies being missed, cannot therefore be excluded. Finally, although the training-pathway barriers motivating this review are described primarily from a UK perspective, they are broadly generalisable to many healthcare systems, with the United States a notable exception given the substantially greater robotic exposure reported during US surgical training [5].

## Conclusion

Technology-enhanced basic robotic skills training demonstrates meaningful promise with evidence supporting improved performance metrics, accelerated learning curves, and objective assessment capabilities. Gaps exist in the utilisation of augmented reality and artificial intelligence for basic robotic skills training and assessment purposes. Moreover, there was lack of enhancement for docking and setup processes alongside the need for standardised assessment frameworks. Adequately powered, multicentre, comparative studies, ideally linked to objective performance metrics and clinical correlation, will be required to evaluate technology-enhanced basic robotics skills learning.

## Supplementary Information

Below is the link to the electronic supplementary material.

Supplementary Material 1

Supplementary Material 2

## Data Availability

No datasets were generated or analysed during the current study.
