# Supplementary material for: Technology-enhanced training in basic robotic surgical skills: a systematic review
Source: J Robot Surg. 2026 Jul 20;20(1):660. doi: 10.1007/s11701-026-03633-w (PMC13384988; doi:10.1007/s11701-026-03633-w)
Supplement: Supplementary file 1 — Supplementary Material 1 [file 11701_2026_3633_MOESM1_ESM.docx]

| **Database:** | **Ovid MEDLINE(R) ALL <1946 to May 20, 2025>** | **Results per line:** | **Number of results: 4641** |
| --- | --- | --- | --- |
| **Date:** | **23/05/2025** |  |  |
| 1 | Robotic Surgical Procedures/ | 18342 |  |
| 2 | Robotics/ | 29222 |  |
| 3 | (robot* or robotic* simulation* or (robotic* adj4 curriculu*) or robotic* rotation* or robotic* training or Robot assisted surg* or Robotic surg* or Surgical robotics or (Surgical skill* assessment adj4 robot*) or (Surgical education adj4 robot*)).ti,ab,kw,kf. | 79851 |  |
| 4 | 1 or 2 or 3 | 85091 |  |
| 5 | Simulation Training/ | 6361 |  |
| 6 | exp Computer Simulation/ | 307025 |  |
| 7 | exp Virtual Reality/ | 6754 |  |
| 8 | Augmented Reality/ | 1497 |  |
| 9 | Computer-Assisted Instruction/ | 12687 |  |
| 10 | exp simulation training/ | 12080 |  |
| 11 | Artificial Intelligence/ | 46014 |  |
| 12 | (TEL or Technology Enhanced Training or simulation* training).ti,ab,kw,kf. | 8660 |  |
| 13 | ((Virtual realit* or Multi-camera system* or augmented realit* or 3D video or 3 D video or artificial intelligence or AI or da vinci or intuitive surgical or CMR surgery or Hugo or Hinotori or online tool*) adj8 train*).ti,ab,kw,kf. | 5902 |  |
| 14 | 5 or 6 or 7 or 8 or 9 or 10 or 11 or 12 or 13 | 378275 |  |
| 15 | 4 and 14 | 5029 |  |
| 16 | editorial/ | 691827 |  |
| 17 | news/ | 225063 |  |
| 18 | exp historical article/ | 411078 |  |
| 19 | anecdotes as topic/ | 4747 |  |
| 20 | case reports/ | 2405685 |  |
| 21 | (letter or comment*).ti. | 199885 |  |
| 22 | (abstract or comment or letter).pt. | 1767588 |  |
| 23 | 16 or 17 or 18 or 19 or 20 or 21 or 22 | 5087335 |  |
| 24 | 15 not 23 | 4799 |  |
| 25 | limit 24 to english language | 4641 |  |

| **Database:** | **Embase <1974 to 2025 Week 20>** | **Results per line:** | **Number of results: 5939** |
| --- | --- | --- | --- |
| **Date:** | **23/05/2025** |  |  |
| 1 | robot assisted surgery/ | 26479 |  |
| 2 | robotics/ | 48719 |  |
| 3 | (robot* or robotic* simulation* or (robotic* adj4 curriculu*) or robotic* rotation* or robotic* training or Robot assisted surg* or Robotic surg* or Surgical robotics or (Surgical skill* assessment adj4 robot*) or (Surgical education adj4 robot*)).ti,ab,kw,kf. | 114654 |  |
| 4 | 1 or 2 or 3 | 126868 |  |
| 5 | exp simulation training/ | 10587 |  |
| 6 | exp Computer Simulation/ | 174406 |  |
| 7 | augmented reality/ | 3120 |  |
| 8 | virtual reality/ | 29090 |  |
| 9 | patient simulation/ | 1871 |  |
| 10 | high-fidelity patient simulation/ | 36 |  |
| 11 | educational technology/ | 3534 |  |
| 12 | artificial intelligence/ | 77849 |  |
| 13 | (TEL or Technology Enhanced Training or simulation* training).ti,ab,kw,kf. | 12436 |  |
| 14 | ((Virtual realit* or Multi-camera system* or augmented realit* or 3D video or 3 D video or artificial intelligence or AI or da vinci or intuitive surgical or CMR surgery or Hugo or Hinotori or online tool*) adj8 train*).ti,ab,kw,kf. | 8249 |  |
| 15 | 5 or 6 or 7 or 8 or 9 or 10 or 11 or 12 or 13 or 14 | 271241 |  |
| 16 | 4 and 15 | 7897 |  |
| 17 | letter/ or case report/ or case study/ | 4080882 |  |
| 18 | (letter or comment*).ti. | 245131 |  |
| 19 | (abstract or letter or editorial or note).pt. | 8269167 |  |
| 20 | 17 or 18 or 19 | 10685319 |  |
| 21 | 16 not 20 | 6192 |  |
| 22 | limit 21 to english language | 5939 |  |

| **Database:** | **Cochrane Central Register of Controlled Trials (CENTRAL) and Cochrane Database of Systematic Reviews (CDSR)** | **Results per line:** | **Number of results: CENTRAL: 820 CDSR: 1** |
| --- | --- | --- | --- |
| **Date:** | **24/05/2025** |  |  |
| #1 | MeSH descriptor: [Robotic Surgical Procedures] this term only | 947 |  |
| #2 | MeSH descriptor: [Robotics] this term only | 1192 |  |
| #3 | (robot* or robotic* simulation* or (robotic* near/4 curriculu*) or robotic* rotation* or robotic* training or Robot assisted surg* or Robotic surg* or Surgical robotics or (Surgical skill* assessment near/4 robot*) or (Surgical education near/4 robot*)):ti,ab | 7463 |  |
| #4 | #1 or #2 or #3 | 7551 |  |
| #5 | MeSH descriptor: [Simulation Training] this term only | 1009 |  |
| #6 | MeSH descriptor: [Computer Simulation] explode all trees | 3968 |  |
| #7 | MeSH descriptor: [Virtual Reality] explode all trees | 1115 |  |
| #8 | MeSH descriptor: [Augmented Reality] this term only | 111 |  |
| #9 | MeSH descriptor: [Computer-Assisted Instruction] this term only | 1484 |  |
| #10 | **MeSH descriptor: [Simulation Training] explode all trees** | 1659 |  |
| #11 | **MeSH descriptor: [Artificial Intelligence] this term only** | 622 |  |
| #12 | (TEL or Technology Enhanced Training or simulation* training):ti,ab | 4141 |  |
| #13 | train* Near/8 (Virtual realit* or Multi-camera system* or augmented realit* or 3D video or 3 D video or artificial intelligence or AI or da vinci or intuitive surgical or CMR surgery or Hugo or Hinotori or online tool*):ti,ab | 29184 |  |
| #14 | #5 or #6 or #7 or #8 or #9 or #10 or #11 or #12 or #13 | 37023 |  |
| #15 | #4 and #14 | 821 |  |
