# Supplementary material for: Technology-enhanced training in basic robotic surgical skills: a systematic review
Source: J Robot Surg. 2026 Jul 20;20(1):660. doi: 10.1007/s11701-026-03633-w (PMC13384988; doi:10.1007/s11701-026-03633-w)
Supplement: Supplementary file 2 — Supplementary Material 2 [file 11701_2026_3633_MOESM2_ESM.docx]

| **Supplementary Appendix S2. GRADE Summary of Findings — Technology-enhanced training adjunct vs standard simulator-based training in basic robotic surgical skills acquisition (novices)** | | | | | | | | |  |  |
| --- | --- | --- | --- | --- | --- | --- | --- | --- | --- | --- |
| *Patient or population: novices in robotic surgery (medical students, surgical residents, fellows, or consultants without prior robotic experience). Intervention: technology-enhanced training adjunct (VR, AR, sensor-based, video review, or performance feedback). Comparator: standard simulator-based training (typically da Vinci Skills Simulator or Fundamentals of Robotic Surgery dry-lab). Setting: 17 studies, predominantly single-centre, all from high-income countries.* | | | | | | | | |  |  |
| **Outcome** | **Number of participants (studies)** | **Risk of bias** | **Inconsistency** | **Indirectness** | **Imprecision** | **Other considerations** | **Effect / Direction** | **Certainty of evidence (GRADE)** | |  |
| **Composite simulator score (overall performance)** | n = 120 (4 RCTs) Feifer 2011; Yang 2017a; Yang 2017b; Takagi 2023 | Not serious | Not serious (e) | Serious (a)(b) | Serious (c) | Publication bias undetected (h) | Technology-enhanced adjuncts associated with statistically significant gains in simulator composite scores compared with standard simulator training. Magnitudes range from a 27.9% improvement in normalised composite score (Feifer 2011) to absolute mean differences of 64→91 vs 63→79 (CESIR; Yang 2017b) and 90.8 vs 72.4 (Takagi 2023). Effect sizes and 95% CIs were not reported by source studies. | **⊕⊕○○ Low** | |  |
| **Time-to-completion (TTC)** | n ≈ 184 (5 RCTs) Chien 2012; Hardon 2022; Eley 2024; Postema 2024; Bechtolsheim 2024 | Not serious | Serious (d) | Serious (a)(b) | Serious (c) | Publication bias undetected (h) | Direction of effect inconsistent across studies. One RCT favours technology (Chien 2012, p=0.002); three show no between-arm difference (Hardon 2022; Eley 2024: 22 vs 22.5 min, p=0.880; Postema 2024). Bechtolsheim 2024 showed faster TTC for console-trained vs VR-trained arm at midpoint (59.2 vs 73.4 s, p=0.007) but not at endpoint. CIs not reported. | **⊕○○○ Very low** | |  |
| **Kinematic precision (path length, instrument velocity, economy of movement)** | n ≈ 104 (3 RCTs, 1 prospective cohort) Chien 2012; Hardon 2022; Judkins 2008; Postema 2024 | Not serious | Serious (d) | Serious (a)(b) | Serious (c) | Publication bias undetected (h) | Direction of effect mixed. Chien 2012: 15.0% improvement in economy of movement vs 0.63% in control (p<0.001). Judkins 2008: AR speed overlay approximately doubled mean instrument velocity (21 to 38 mm/s, p<0.05), retained at 2 weeks. Hardon 2022: no between-arm path-length difference. Postema 2024: reduced dominant-hand collisions (8.73 vs 14.27, p=0.045). | **⊕○○○ Very low** | |  |
| **Force application during suturing/dissection** | n = 125 (2 RCTs, intervention) Hardon 2022; Bechtolsheim 2024 (plus Rahimi 2023, n=60, construct-validity — not pooled (g)) | Not serious | Not serious (e) | Serious (a)(b) | Serious (c) | Publication bias undetected (h) | No demonstrated advantage of technology-enhanced training over standard simulator training. Hardon 2022: no difference in force application after training. Bechtolsheim 2024: both VR and live-console arms reduced peak force from baseline; no significant final difference (suturing peak force 4.1 N vs 4.7 N, p=0.086). Rahimi 2023 supports force as discriminative of expertise but does not address intervention effectiveness. | **⊕⊕○○ Low** | |  |
| **Learning curve / repetitions to proficiency** | n = 107 (2 RCTs) Takagi 2023; Bechtolsheim 2024 | Not serious | Not serious (e) | Serious (a)(b) | Serious (c) | Publication bias undetected (h) | Technology-enhanced approaches associated with accelerated learning. Takagi 2023: CUSUM plateau at 2 vs 4 cycles. Bechtolsheim 2024: fewer repetitions to proficiency for the live-console-trained arm vs VR-trained arm on Ring Tower Transfer (2.48 vs 5.45, p<0.001), Knot Tying (5.34 vs 8.13, p=0.006), and Vessel Energy Dissection (2.0 vs 2.38, p=0.001). Direction of advantage varies by adjunct (educational video vs live console training). | **⊕⊕○○ Low** | |  |
| **Validated rating-tool score (R-OSATS / GEARS)** | n = 11 (1 randomised crossover) Nathan 2023 | Not serious | Cannot assess (f) | Not serious | Very serious (c) | Publication bias undetected (h) | VR-classroom training associated with significantly higher R-OSATS scores than self-directed FRS learning (44.80 vs 35.33 points, p=0.006). Single small study; reproducibility unknown. No GEARS-based intervention study was identified. | **⊕○○○ Very low** | |  |
| **Skill retention (≥ 2 weeks post-training)** | n = 30 (1 prospective cohort) Judkins 2008 | Not serious | Cannot assess (f) | Serious (a)(b) | Serious (c) | Publication bias undetected (h) | AR-speed-overlay-trained participants retained kinematic-velocity gains at 2-week post-training assessment (Judkins 2008). No other included study reported a retention test, leaving the durability of training gains across modalities essentially unknown. | **⊕○○○ Very low** | |  |
| **User acceptability / cognitive workload (Likert, NASA-TLX)** | n = 22 (2 prospective cohorts; non-comparative) Casas-Yrurzum 2023; Wu 2021 | Serious (f) | Cannot assess (f) | Serious (a) | Serious (c) | Publication bias undetected (h) | Usability evaluations indicate favourable user-acceptability ratings (Casas-Yrurzum 2023: median Likert 6–7 of 7 across all domains, p<0.001 vs neutral). EEG/eye-tracking-derived cognitive-load metrics predicted task performance with 72.5% accuracy (Wu 2021). No comparative intervention data. | **⊕○○○ Very low** | |  |
| **Operative-skill transfer or patient-level outcomes** | No studies | — | — | — | — | — | No included study reported operative-performance transfer or patient-level outcomes. The effect of technology-enhanced basic-robotic-skills training on downstream clinical performance is therefore unknown from this evidence base. | **No evidence available** | |  |
| **Explanations / footnotes** | | | | | | | | | | |
| *(a) Most included studies enrolled medical students; downgraded for reduced generalisability of population.* | | | | | | | | | | |
| *(b) All outcomes were measured on simulators or in controlled task environments; no included study reported operative-skill transfer or patient-level outcomes. Downgraded for indirectness of outcome* | | | | | | | | | | |
| *(c) Sample sizes are uniformly small (median n=20, range 7–87) and 95% confidence intervals are not reported by source studies; precision of effect estimates therefore cannot be characterised. Downgraded for imprecision.* | | | | | | | | | | |
| *(d) Direction of effect is inconsistent across studies; magnitudes vary substantially. Downgraded for inconsistency.* | | | | | | | | | | |
| *(e) Direction of effect is broadly consistent across studies; magnitude varies but in a manner that does not alter the qualitative conclusion. Not downgraded.* | | | | | | | | | | |
| *(f) Only one study (or non-comparative data) contributes to this outcome; consistency cannot be assessed and the result should be regarded as exploratory.* | | | | | | | | | | |
| *(h) Publication bias could not be formally assessed given the novelty of the technologies and under-reporting of null findings, the possibility of publication bias cannot be excluded but was reported as undetected.* | | | | | | | | | | |
| **GRADE methodology** | | | | | | | | | | |
| *GRADE certainty of evidence was rated at the outcome level using the five downgrading domains (risk of bias, inconsistency, indirectness, imprecision, publication bias) per Schunemann et al. (GRADE Handbook, 2013). Starting certainty was high for randomised studies and low for non-randomised studies. Upgrading factors (e.g. large effect, residual confounding favouring no effect) were not applied to any outcome row, as no included study reported effect.  Symbols: ⊕⊕⊕⊕ high, ⊕⊕⊕○ moderate, ⊕⊕○○ low, ⊕○○○ very low.  Abbreviations: AR, augmented reality; CESIR, Controller of Events on Simulator and Robot; CUSUM, cumulative sum; dVSS, da Vinci Skills Simulator; FRS, Fundamentals of Robotic Surgery; GEARS, Global Evaluative Assessment of Robotic Skills; NASA-TLX, NASA Task Load Index; R-OSATS, Robotic Objective Structured Assessment of Technical Skills; TTC, time-to-completion; VR, virtual reality.* | | | | | | | | | | |
